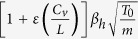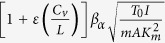# Flutter Phenomenon in Flow Driven Energy Harvester–A Unified Theoretical Model for “Stiff” and “Flexible” Materials

**DOI:** 10.1038/srep35180

**Published:** 2016-10-14

**Authors:** Yu Chen, Xiaojing Mu, Tao Wang, Weiwei Ren, Ya Yang, Zhong Lin Wang, Chengliang Sun, Alex Yuandong Gu

**Affiliations:** 1 Institute of High Performance Computing, Agency for Science, Technology and Research, 1 Fusionopolis Way, #16-16 Connexis, 138632, Singapore; 2 International R&D center of Micro-nano Systems and New Materials Technology, Key Laboratory of Optoelectronic Technology & Systems, Ministry of Education, Chongqing University, Chongqing 400044, P.R. China; 3 Institute of Microelectronics, Agency for Science, Technology and Research, 2 Fusionopolis Way, #08-02 Innovis Tower, 138634, Singapore; 4 Electrical and Computer Engineering, National University of Singapore, 4 Engineering Drive 3, 117583, Singapore; 5 DHI Water & Environment (S) Pte Ltd, 1 Cleantech Loop, #03-05 CleanTech One, 637141, Singapore; 6 Beijing Institute of Nanoenergy and Nanosystems, Chinese Academy of Sciences, Beijing 100083, P.R. China; 7 School of Materials Science and Engineering, Georgia Institute of Technology, Atlanta, Georgia 30332, USA

## Abstract

Here, we report a stable and predictable aero-elastic motion in the flow-driven energy harvester, which is different from flapping and vortex-induced-vibration (VIV). A unified theoretical frame work that describes the flutter phenomenon observed in both “stiff” and “flexible” materials for flow driven energy harvester was presented in this work. We prove flutter in both types of materials is the results of the coupled effects of torsional and bending modes. Compared to “stiff” materials, which has a flow velocity-independent flutter frequency, flexible material presents a flutter frequency that almost linearly scales with the flow velocity. Specific to “flexible” materials, pre-stress modulates the frequency range in which flutter occurs. It is experimentally observed that a double-clamped “flexible” piezoelectric P(VDF-TrFE) thin belt, when driven into the flutter state, yields a 1,000 times increase in the output voltage compared to that of the non-fluttered state. At a fixed flow velocity, increase in pre-stress level of the P(VDF-TrFE) thin belt up-shifts the flutter frequency. In addition, this work allows the rational design of flexible piezoelectric devices, including flow-driven energy harvester, triboelectric energy harvester, and self-powered wireless flow speed sensor.

Harnessing flow/wind kinetic energy to generate electricity has seen a surge in recent years. For example, flow-driven piezoelectric, triboelectric, electromagnetic energy harvesters have attracted much attention^1^,^2^,^3^,^4^,^5^,^6^. To maximize output power density and reduce device cost, common desirable features from above technologies are: 1. Maximum vibration amplitude; 2. Predictable/tunable vibration frequencies; 3. Flexible electricity-generating element.

There are several aero-vibration phenomena that give rise to the dynamic response of the piezoelectric or triboelectrical material vibrating structure under wind loading, including flapping, vortex induced vibration (VIV), and flutter. Flapping is an interacting phenomenon of bending of planar bodies and steady flows, which is observed in flags and can be utilized as energy harvesting device in flapping flag^4^,^7^. However, if the flow velocity increases up to a high level, the flapping can enters a chaotic status^8^. Vortex induced vibrations (VIV)^9^,^10^ are normally observed on deepwater risers in offshore engineering and heat exchanger tubes. While the vortex is shed from the bluff body, there is periodic force applying on the body. If the vortex shedding frequency is close to the natural frequency of the structure/body (“lock” region), there is resonance phenomenon and the amplitude increase dramatically (but not divergent). Thus, the frequency of it is determined by the vortex except the “lock” region.

Different from other aero-vibrations, flutter is a “self-feeding” vibratory motion that results from the coupling of aerodynamic forces with the elastic deformation of a structure^11^. It is often the result of combined bending and torsion and affects plate-like structures, such as signboards and suspension bridge decks. The instability is caused when there is a “positive feedback” between the structure’s natural vibration and the aerodynamic forces. In other words, the movement of the object increases the aerodynamic load, which in turn drives the object to move further. This is why flutter features the largest vibration amplitude amongst all four aero-vibrations mentioned above. When the energy input surpass the mechanical damping of the object, the vibration often results in large divergent amplitude and can lead to catastrophic failure^11^,^12^,^13^,^14^, e.g. 1940 Tacoma Narrow Bridge. This type of catastrophic failure literally excludes the utilization of flutter phenomenon in above mentioned applications for “stiff” materials.

Polymeric (“flexible”) materials have shown to withstand repeated large deformation without suffering non-elastic failure. Hence, flexible materials are great candidates for utilizing flutter phenomenon, compared to “stiff” materials. Although extensively-studied for “stiff” materials, flutters associated with flexible materials are not reported.

In this paper, we introduced a unified theoretical model to quantitatively and qualitatively describe the flutter phenomena associated with both “stiff” and “flexible” material based thin belts. Figure 1 shows the constant flutter frequency for “stiff” materials, compared with different phenomena for “flexible” materials, including the nonlinear effect of belt deformation on the flutter frequency (higher air velocity inducing higher frequency). In addition, the vibration frequency in fluttering is stable in a wide range of air inflow velocity, which is against the phenomenon found in VIV that the vibrating frequency 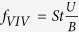 almost linearly increases with air velocity *U* and meanwhile the vibration becomes unstable out of the narrow “lock” region^10^ (see Fig. 1). In order to study the flutter of thin belt under blowing air, an unified theory of flutter for “stiff” and “flexible” material based belts is established as following.

## Results

Two different materials were employed in this study to investigate the flow driven energy harvester: “Stiff” material-AlN/Si vibratory micro-belt and “flexible” material- P(VDF-TrFE) micro-belt. The Aluminum Nitride (AIN) is a famous and promising popular piezoelectric functional material in MEMS energy harvesting technology, and it works well under harsh environment applications due to its high Curie temperature as 1150 °C. Besides, silicon (Si) is commonly used as the substrate materials in semiconductor related industry and research, thus such AlN-Si bimorph structure is chosen as the “stiff material” in the current study. On the other hand, P(VDF-TrFE) is chosen as the “flexible” material in this study because of its unique properties: 1. potential applications on curved surface due to flexibility; 2. high mechanical strength; 3. dimensional stability; 4. high and stable piezoelectric coefficient up to approximately 90; 5. chemical inertness; 6. thickness of the film can be customized from *μ*m to mm.

**Figure 2:**
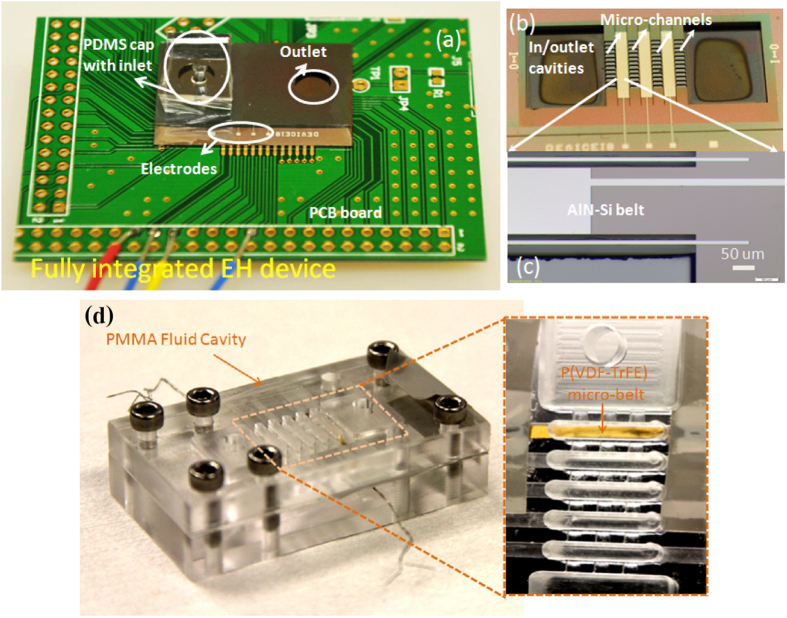
(**a**) Fully integrated “stiff” material AlN-Si micro-belt based energy harvester device; (**b**) the inner functional structure of AlN-Si micro-belt based energy harvester device; (**c**) the zoom-in image of the AlN-Si micro-belt; (**d**) fully integrated “flexible” material P(VDF-TrFE) micro-belt based energy harvester device, inset is the zoom-in image of the P(VDF-TrFE) micro-belt.

Fully integrated “stiff” material AlN-Si and “flexible” material P(VDF-TrFE) micro-belt based energy harvester device are presented in Fig. 2. The detailed fabrication methods are introduced in §1 of Supplementary Information.

MSA-500 Micro System Analyzer is utilized to measure the “stiff” and “flexible” material vibratory belt structures by removing the top cap of the device. This equipment can quickly identify, visualize and measure system resonances and transient responses on small structures by utilizing the technology solutions like ESPI, white light interferometry, and phase-shifting interferometry. Using wide-band excitation, the highly sensitive laser-Doppler technique can rapidly find all mechanical resonance (in-plane and out-of-plane) without prior information. In a second step, stroboscopic video microscopy is used to obtain accurate amplitude and phase information of in-plane resonances identified through laser vibrometry.

**Figure 3:**
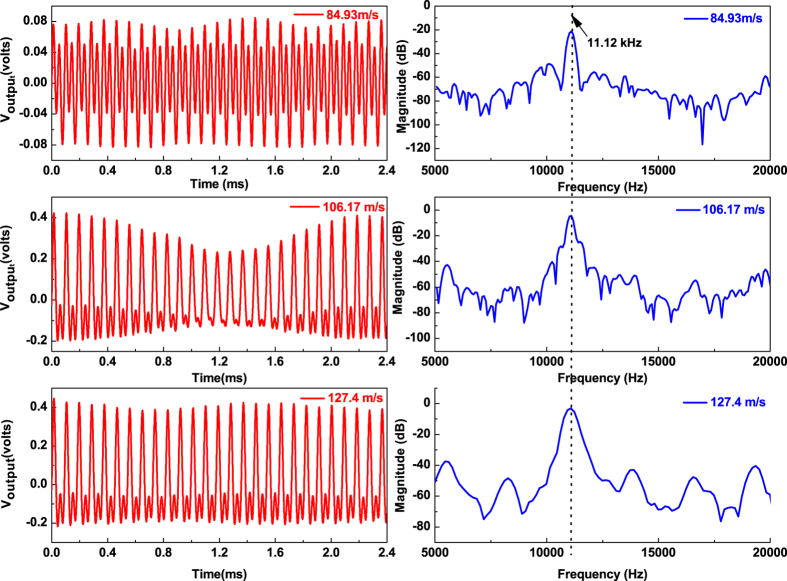
The measured time and frequency domain of the electrical signal of the double clamped “stiff” material AlN/Si based micro-belt (length × width × thickness: 10 mm × 1 mm × 21 *μm*) at the different inlet air flow velocity of 84.93 m/s, 106.17 m/s, and 127.4 m/s.

**Figure 4:**
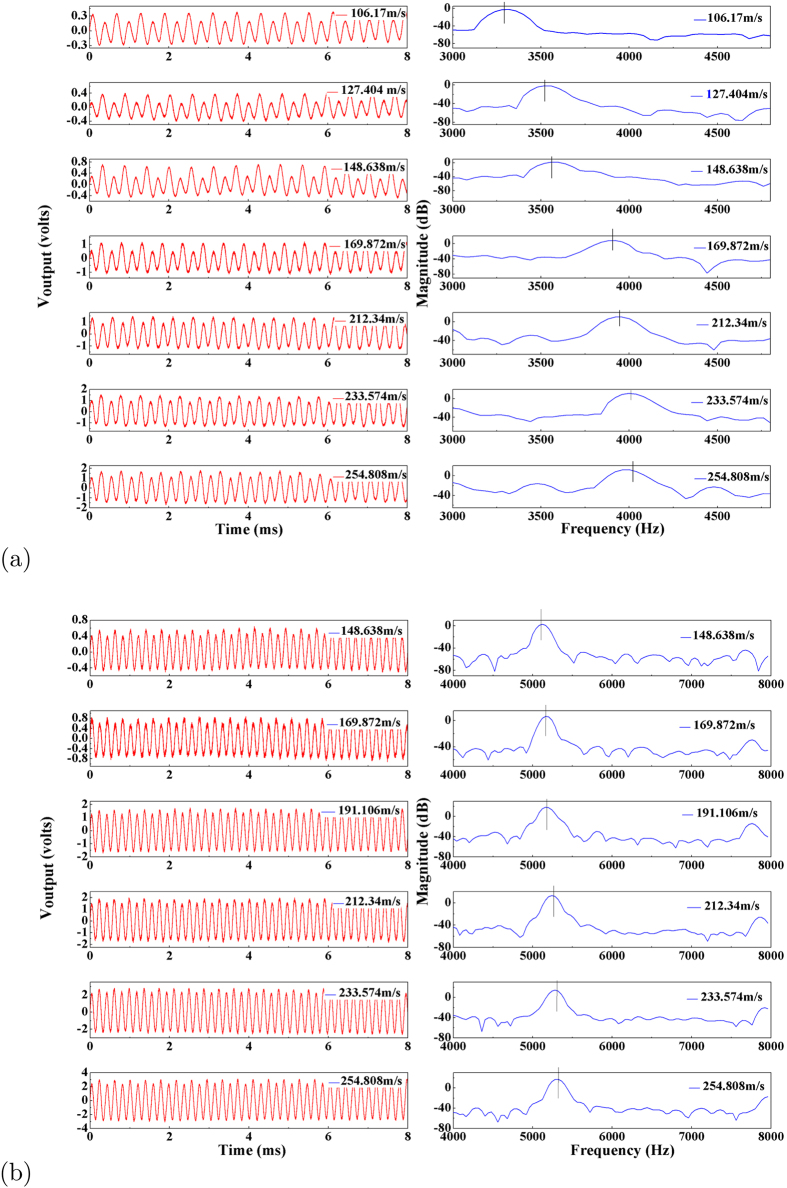
The measured time and frequency domain of the electrical signal of the double clamped “flexible” material P(VDF-TrFE) based micro-belt (length × width × thickness: 22 mm × 1.2 mm × 20 *μm*) at different inlet air flow velocity under two prestress conditions: (**a**) *T*_0_ = constant ≈ 0.07 N and (**b**) 0.21 N.

**Figure 5:**
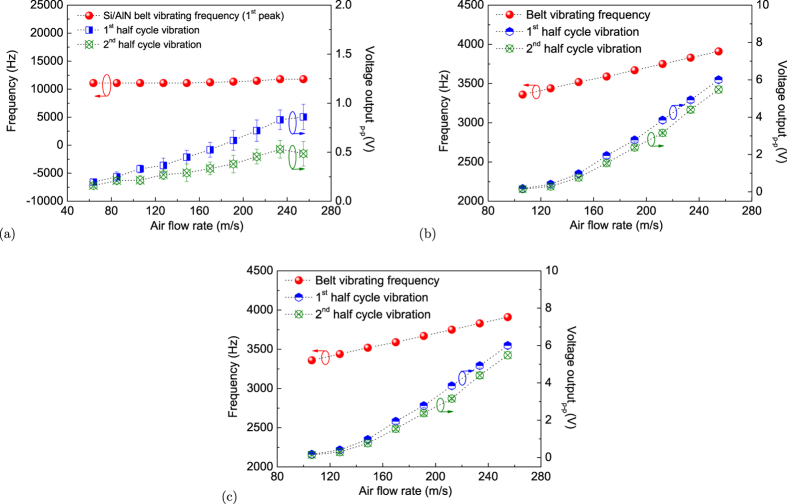
The measured dominant frequency and voltage output of double-clamped belt vs. air flow velocity (**a**) AlN + Si (length × width × thickness: 10 mm × 1 mm × 21 *μm*); (**b**) less pre-stressed P(VDF-TrFE) (length × width × thickness: 22 mm × 1.2 mm × 20 *μm*) under prestress *T*_0_ = constant ≈ 0.07 N; (**c**) more pre-stressed P(VDF-TrFE) (length × width × thickness: 22 mm × 1.2 mm × 20 *μm*) under prestress *T*_0_ = constant ≈ 0.21 N.

In the experiment, it is observed as shown in Fig. 3 that there is a critical air flow velocity, below which the output voltage is extremely small (<0.05 mV), while above which the output voltage suddenly jumps to a high level (0.04 V). Similar phenomenon can also be found for “flexible” material based belt from its voltage output as shown in Fig. 4. The combination of the bending and torsion vibrations observed in the experiment confirms the occurrence of fluttering phenomena. It is also noticed that the dominant frequency for “stiff” materiel keeps almost as a constant, while that for “flexible” material scales with the air inflow velocity as shown in Fig. 4, and such phenomenon can be clearly seen from Fig. 5.

## Discussion

To further illustrate the occurrence of fluttering and its characteristics, theory was developed and employed to predict the onset velocity and fluttering frequency at different flow rate in the next section. The distinct behaviors for belts based on different materials, which is associated to the correlation between flutter frequency and the deformation amplitude, is also discussed in depth herein.

As known, flutter is a complex combination of bending and torsional vibration modes which cross-feed each other, resulting in divergent vibration amplitude^11^. It is characterized by an onset air flow velocity *U*_*onset*_, below which flutter does not occur. In addition, flutter normally occurs when natural torsion frequency *f*_*α*0_ is higher than natural bending frequency *f*_*h*0_, and the flutter frequency *f*_*F*_ is slightly lower than the natural torsion frequency^11^,^12^,^13^,^14^ (see §2 of Supplementary Information).

For natural vibration of “stiff” belts which is governed by stiffness, the theoretical model has already been established well^15^,^16^; and the theoretical model for bending vibration of pre-stressed “flexible” belts/string is also widely used under conditions with small amplitude^17^,^18^ and large amplitude^19^,^20^,^21^,^22^,^23^. However, there is a lack of model for torsional motion of “flexible” belts with pre-stress and finite vibration amplitude are missing. So, it is important to establish an unified model for the bending and torsional vibrations for “stiff” and “flexible” belts with pre-stress and finite vibration amplitude which is possibly observed in the real world applications (see §2 of Supplementary Information):


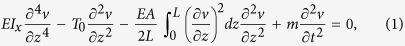



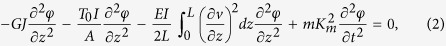


where *x* is along the flow direction, *z* is along the axis of beam or belt, *y* is the normal direction of the belt surface, and *t* is time. *v* is the deformation of the belt along the *y* direction, and *φ* is the torsional angle of each element of the belt. The following are the belt materials properties. *I*_*x*_ is the second axial moment of area about the x-direction. *E* and *G* are the Young’s and shear modules, *T* is the axial internal force, and *m* is the mass per unit length, *J* is the torsional constant (stiffness), *K*_*m*_ is the radius of gyration, *A* is the cross section area (see §2 of Supplementary Information).

Accordingly, the natural bending and torsion frequency for belts with different material under different vibrating situation can be analyzed as following. Especially, for the double clamped belts of “stiff” material, the axial internal force *T*_0_ and the vibration amplitude *C*_*v*_ are small due to its high stiffness, leading to a result that the second term and the third (nonlinear) term in Eqs (1) and (2) are relatively small. On the other hand, for the double clamped belts of “flexible” material, the stiffness of the material is much smaller than the effect of axial internal force *T*_0_, thus the first terms in Eqs (1) and (2) are ignorable, while the third terms (nonlinear) are un-neglectable when bending amplitude *C*_*v*_ is large. So theoretically, it can be summarized that the natural frequency of “stiff” material is almost constant, while the frequency of “flexible” material increases with increasing bending amplitude, which is presented in Table 1 (see Eqs S1.1 and S1.2 of Supplementary Information).

To verify the above theory about flutter phenomena for long and thin belt (length ≫ width ≫ thickness) in air flow, the experimental results are studied in details and depth. First, the linear flutter theory is validated by examining the motion of “Stiff” material belt which is easy to surpass the material mechanical limit (fracture strength) at high air flow velocity due to the ultra-low toughness and stress concentration at the belt’s ends. It is found in the experiment that the maximum bending deformation of AlN/Si belt is around 0.015 mm (see §3 of Supplementary Information) before it cracks, corresponding to 1–2 degrees of maximum torsion (see Eq. S1.26 of Supplementary Information). Within such small bending deformation magnitude (≈0.01 mm, see §3 of Supplementary Information), the natural vibration frequencies for “stiff” belt almost remain as constants when bending deformation is increasing (see §4 of Supplementary Information). Thus, the linear Euler-Bernoulli theory for bending and St. Venant theory for torsion^15^,^16^.


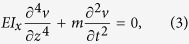



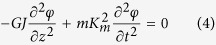


are valid for fluttering “stiff” material micro belts.

**Figure 6:**
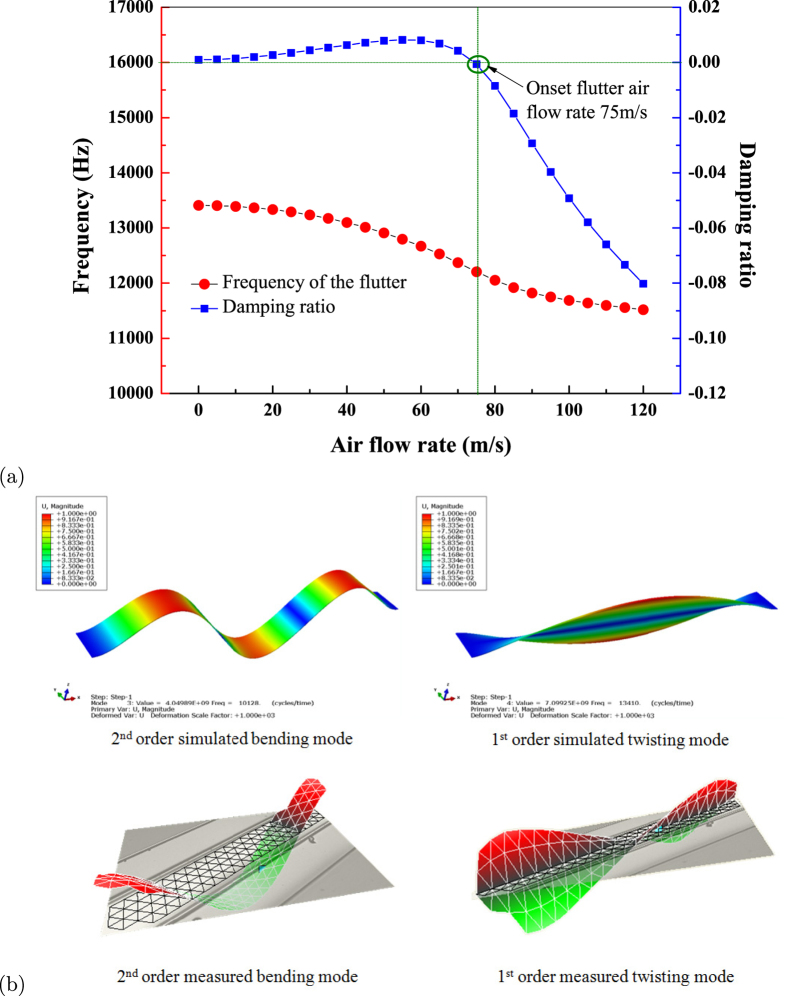
(**a**) The theoretically deduced condition of the flutter occurrence (air flow velocity vs. flutter frequency and damping ratio), the critical points where the daming ratio becomes negative indicate the air flow velocity corresponding the onset of flutter 75 m/s; (**b**) the two dominant vibration modes of the fluttering for double clamped “stiff” material AlN/Si based belt measured by Laser Doppler compared with vibration modes obtained by FEM.

Based on Selberg and Theodorsen’s theories that flutter occurs when bending and torsional modes are “self-feeding”, pairs of bending and torsional modes are analyzed theoretically to investigated the possibility of flutter (see §2 of Supplementary Information). It is theoretically revealed in Fig. 6(a) that flutter with 3rd order bending (10.128 kHz, based on the finite element method simulation results by ABAQUS software) with 1st order torsional (13.410 kHz) modes becomes onset when air velocity is larger than 75 m/s, which is defined as *U*_*onset*_. The experimental measurement of highly sensitive Laser-Doppler technique (MSA-500 Micro System Analyzer) shows very obvious existence of such two vibration modes for fluttering thin belt in inflow air, which is presented together with the finite element method simulation results in Fig. 6(b).

**Figure 7:**
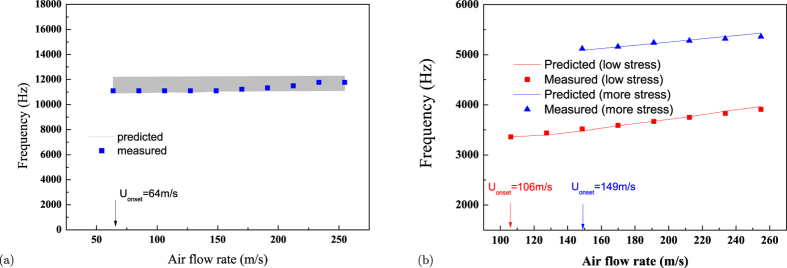
The measured and theoretically predicted flutter frequency vs. air flow velocity above the flutter onset velocity for (**a**) double-clamped AlN/Si belt (length × width × thickness: 10 mm × 1 mm × 21 *μm*), and (**b**) for P(VDF-TrFE) (length × width × thickness: 22 mm × 1.2 mm × 20 *μm*) under two prestress conditions (*T*_0_ = constant ≈ 0.07 N and 0.21 N). The uncertainty of theoretically predicted data for “stiff” belt is presented in area filled in grey color, where such uncertainty comes from the difference between individual AlN/Si belt. The data indicate that the flutter frequency is rarely constant for the “stiff” material based belt as the air flow velocity increases, while the frequency scales almost linearly with the air flow velocity, and flutter frequency are modulated by prestress for “flexible” material.

Figure 7(a) shows the theoretically predicted dynamic behavior together with the measured phenomenon for the double-clamped “stiff” material AlN/Si based micro-belt, which is investigated through monitoring the piezoelectric voltage output of the belt. Experimentally, low air flow velocity does not initiate efficient vibration of the double-clamped “stiff” AlN/Si micro-belt (voltage output is extremely low), but this vibration becomes suddenly periodical and stable when the air flow velocity exceeds a critical value 64 m/s, which agrees very well with the theoretical prediction of flutter onset velocity as 75 m/s. Considering the minor difference of each individual micro belt, the observation that the dominant frequencies of the vibration is almost independent on air velocity as 11.12 kHz quantitatively aligns well to the predicted flutter frequency which remains relatively constant as the air flow velocity increases above *U*_*onset*_.

In contrast of “stiff” material AlN/Si, the “flexible” material has relatively low stiffness but its high toughness ensures it to bend and twist with large deformation. So the deformation can increase up with the air velocity until the angle of attack roughly reaches the stall angle (normally 30–45 degrees for thin film plate^8^,^24^) due to the lack of aerodynamic lift and torque.

As seen from the governing equations of vibration for belts (see Eqs 1 and 2), the bending deformation has nonlinear effects on the natural vibration frequency when the deformation amplitude *C*_*v*_ is large where linear theories^15^,^16^ are invalid. In contrast of “stiff” material, the vibration of “flexible” material is dominated by the internal axial stress within the belt. As known, the higher the internal axial pre-stretching force *T*_0_ is, the higher the vibration frequency 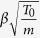 (see Table 1) will be; which can be found in adjustment of strings of piano and violin. Similarly, in the current study, large bending deformation elongates the axial length of the belt by an extra strain 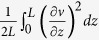, which increases the total axial force up to 
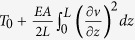
. Thus, it can be implied that the increase in both pre-stress and bending deformation amplitude *C*_*v*_ rises the equivalent effective internal axial force 
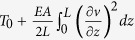
, then resulting in a raise in the natural vibration frequency. Finally, the increase in natural vibration frequency induces higher flutter frequency, which is almost determined by the torsional frequency.

A detailed numerical simulation (see §4 of Supplementary Information) was conducted to quantitatively evaluate this nonlinear effect on flutter frequency of “flexible” P(VDF-TrFE) micro belt in the current experiment with the bending deformation amplitude ranging within 0–0.4 mm (estimated based on voltage output, see §3 of Supplementary Information). The theoretically predicted flutter frequency of P(VDF-TrFE) micro belt is presented in Fig. 7(b) together with experimental measurement against the incoming air velocity and bending deformation amplitude *C*_*v*_ at two different axial pre-stretching force *T*_0_ = constant ≈ 0.07 N and 0.21 N. Such bending deformation (up to 0.4 mm) is magnified compared to that of “stiff” material as 0.015 mm (see §3 of Supplementary Information). The theoretical prediction shows that the flutter frequency increases from 3300 Hz to 3800 Hz and 5050 Hz to 5375 Hz with the increasing bending deformation amplitude (incoming air velocity) for less and more pre-stressed “flexible” micro belt, which agrees well with the experimental finding. Furthermore, the increasing pre-stress makes the flutter frequency of “flexible” belt to become higher at the same air velocity, which is also the modulation effect predicted by the theory. Thus, it can be concluded that the flutter frequency of “flexible” material is sensitive to the air velocity, and the pre-stress can be utilized to modulate the flutter frequency.

## Conclusion

A unified theoretical frame work that describes the flutter of both “stiff” and “flexible” materials are established and experimentally validated in this study. A steep rise in vibration amplitude of double-clamped belt characterizes the flutter phenomenon observed in both materials, due to the coupled effects of the bending and torsional modes of the belt. The observation that higher incoming air velocity induces higher vibration amplitude for both materials also agrees well with the unified analytical model.

The flutter frequency of “stiff” materials is found independent of the air flow velocity. Catastrophic failures were observed for “stiff” materials when air flow exceeds the rate at which flutter first occurs. In comparison, no such failure is observed for “flexible” material in the flutter regime, allows full utilization of the maximum displacement associated with flutter. Flutter frequency scales near linearly with flow velocity for the “flexible” material. The frequency range over which flutter occurs can be modulated by pre-stress. “Flexible” piezoelectric vibrating belt employing flutter phenomenon can be rationally designed as a sensing element to detect and monitor the pneumatic flow velocity in a flow field. Tuning the frequency through pre-stress of the “flexible” piezoelectric belt also allows fitting the energy harvester to the power management IC for maximum energy harvesting efficiency.

## Additional Information

**How to cite this article**: Chen, Y. *et al.* Flutter Phenomenon in Flow Driven Energy Harvester–A Unified Theoretical Model for ‘‘Stiff’’ and ‘‘Flexible’’ Materials. *Sci. Rep.* **6**, 35180; doi: 10.1038/srep35180 (2016).

## Supplementary Material

Supplementary Information

## Figures and Tables

**Figure 1:**
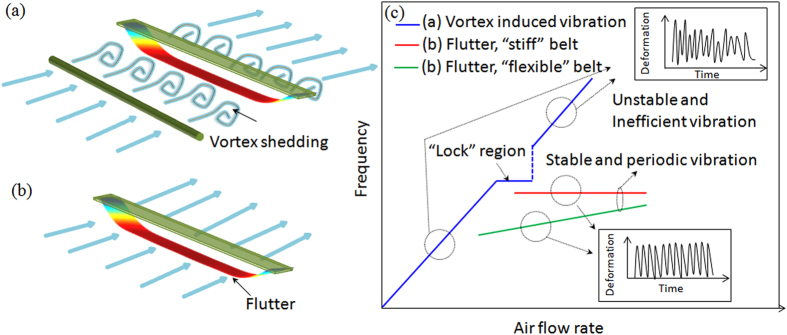
The schematic of the (**a**) vortex induced vibration; (**b**) flutter phenomenon; and the (**c**) the summary of relationship between the air flow velocity and the frequency of the vibrating belt under vortex shedding effect, “stiff” belt vibrating under flutter effect and the “flexible” belt vibrating under flutter effect; the insets are the deformation behavior of the above three condition.

**Table 1:** Theoretical predicted frequencies of natural vibration in different modes, *β*_*h*_ and *β*_*α*_ are determined by boundary condition at two ends of a belt and vibration mode number, *ε* is related to the maximum normalized bending deformation amplitude *C*_*v*_/*L*.

Material	Bending *f*_*h*0_	Torsion *f*_*α*_0
“stiff” belt	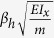	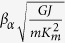
“flexible” belt with small deformation	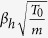	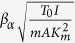
“flexible” blet with large deformation