# Supplementary material for: Flutter Phenomenon in Flow Driven Energy Harvester–A Unified Theoretical Model for “Stiff” and “Flexible” Materials
Source: Sci Rep. 2016 Oct 14;6:35180. doi: 10.1038/srep35180 (PMC5064364; doi:10.1038/srep35180)
Supplement: Supplementary Information [file srep35180-s1.pdf]

Supplementary Information: Flutter Phenomenon in Flow Driven Energy Harvester–A Unified Theoretical Model for “Stiff” and “Flexible” Materials

Yu Chen[1,2], Xiaojing Mu[*2,3], Tao Wang[4], Weiwei Ren[5], Ya Yang[†6], Zhong Lin Wang[6,7], Chengliang Sun[3] and Alex Yuandong Gu[‡3]

[1] *Institute of High Performance Computing, Agency for Science, Technology and Research, 1 Fusionopolis Way, #16-16 Connexis, Singapore 138632*
[2] *International R&D center of Micro-nano Systems and New Materials Technology, Key Laboratory of Optoelectronic Technology & Systems, Ministry of Education, Chongqing University, Chongqing 400044, P.R. China*
[3] *Institute of Microelectronics, Agency for Science, Technology and Research, 2 Fusionopolis Way, #08-02 Innovis Tower, Singapore 138634*
[4] *Electrical and Computer Engineering, National University of Singapore, 4 Engineering Drive 3, Singapore 117583*
[5] *DHI Water & Environment (S) Pte Ltd, 1 Cleantech Loop, #03-05 CleanTech One, Singapore 637141*
[6] *Beijing Institute of Nanoenergy and Nanosystems, Chinese Academy of Sciences, Beijing 100083, P.R. China*
[7] *School of Materials Science and Engineering, Georgia Institute of Technology, Atlanta, Georgia 30332, USA*

ABSTRACT

This Supplementary Information provides the experimental configuration, detailed theory about flutter phenomena, the relationship between the deformation and voltage output of microbelts, and the effect of microbelts’ vibration deformation on flutter frequency.

S1 Experimental setup and phenomexna

“Stiff” material-AlN/Si vibratory micro-belt: A 3-wafer bonding CMOS compatible process was utilized. Key process integration steps are shown in Figs. S1(a)–(h). The process start with a functional AlN/Mo/AlN stacks (Figure S1(a)) deposited on a silicon on isolation (SOI) wafer; 0.7 μm thick Al was then deposited as electrical pad material. After that, two masks (Figs. S1(b)–(d)) were used for top Al, bottom Mo electrodes patterning and top side release etching to define the device layer. Subsequently, bottom side of the device wafer was patterned and partial etched to define the cavity (Figure S1(e)). At the same time, the top cap wafer was processed to define the top cavity and the inlet and outlet hole (Figure S1(f)). Then the device wafer was bonded to the top cap wafer and the bottom side of the device layer was released. Finally, a bare Si wafer was bonded to the bottom side of device wafer to seal the cavity, which also acts as the support wafer for the top side inlet and outlet hole release (Figure S1(h)).

“flexible” material- P(VDF-TrFE) micro-belt: To characterize the aerodynamic behavior of the “flexible” material based micro-belt, P(VDF-TrFE) was select as the vibratory film, the thickness of which is 20 μm and the planar size is 10 mm $\times$ 1 mm. The top and bottom of the P(VDF-TrFE) micro-belt is coated by 400 Å Ti/Au as electrodes. Differ with the fabrication process of the “stiff” material, the fluid channel cavity was realized by precision machined on the PMMA material. The “flexible” material based micro-belt is placed in the groove of the PMMA case and then fixed tightly on both sides by threaded connection between the top cap and the bottom case body.

The Piezoelectric and mechanical property of the AlN and P(VDF-TrFE) are summarized in Tab. S1.

The schematic of the dominant frequencies, the corresponding voltages and powers measurement set-up is shown in Figure S2. The flow source is compressed air; a mass flow controller (ALICAT) was connected in series with the flow source and the “stiff” and “flexible” material vibratory belt based EHs that are under tests. A Digital Phosphor Oscilloscope (Tektronix DPO 7354) was employed to read out the dominant electrical signal frequencies and the corresponding voltages of the “stiff”

*Corresponding author Email: mxjacj@cqu.edu.cn
†Corresponding author Email: yayang@binn.cas.cn
‡Corresponding author Email: agupersonal@gmail.com

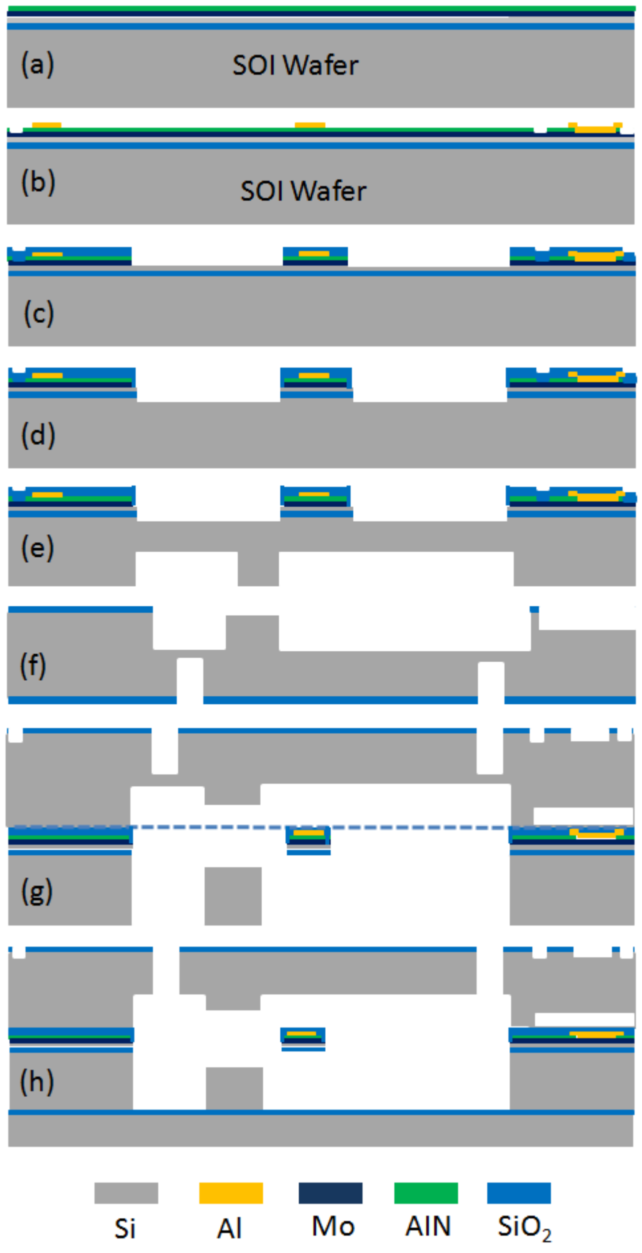

Figure S1. Major process integration steps. (a) The piezoelectric 0.02 μm AlN /0.2 μm Mo/1.2 μm AlN stack were consecutively deposited on a SOI wafer; (b) Pattern the top and bottom Al electrodes; (c) Pattern the piezoelectric stack; (d) 20 μm Si+ 1 μm SiO_2+ 80 μm Si etch + Backside wafer Grinding to 550 μm; (e) Backside 250 μm partial Si etch; (f) Top cap wafer fabrication; (g) bonding top cap wafer with the device wafer and (h) Inlet and outlet hole forming by DRIE on top cap wafer, after that bonding bottom wafer to device wafer and final partial dicing to expose top electrodes.

Table S1. The piezoelectric and mechanical properties of the "stiff" material AlN, "flexible" material P(VDF-TrFE) and silicon.

	AlN	P(VDF-TrFE)	Si
d_{31}	-1.9 (pC/N)	6 ± 20% (pC/N)	-
d_{33}	5 (pC/N)	-20 ± 20% (pC/N)	-
E	329 GPa	3.5 GPa (machine direction)	185 Gpa
		3.5 GPa (transverse direction)	
ε_{11}	8.0(10^{-11}F/m)	10.18(10^{-11}F/m)	-
ε_{33}	9.5(10^{-11}F/m)	10.18(10^{-11}F/m)	-
ρ	3260 kg/m^3	1800 kg/m^3	2331 kg/m^3
ν	0.24	0.18	0.278

and "flexible" material based vibratory belt; and the powers can be calculated by parallel connecting a external resistor to the device under test.

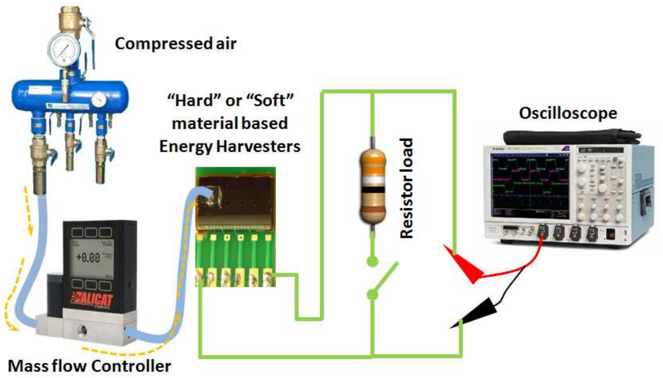

Figure S2. The schematic measurement setup of the dominant frequencies, the corresponding voltages, and the powers generation on the "stiff" or "flexible" material vibratory micro-belt based EHs.

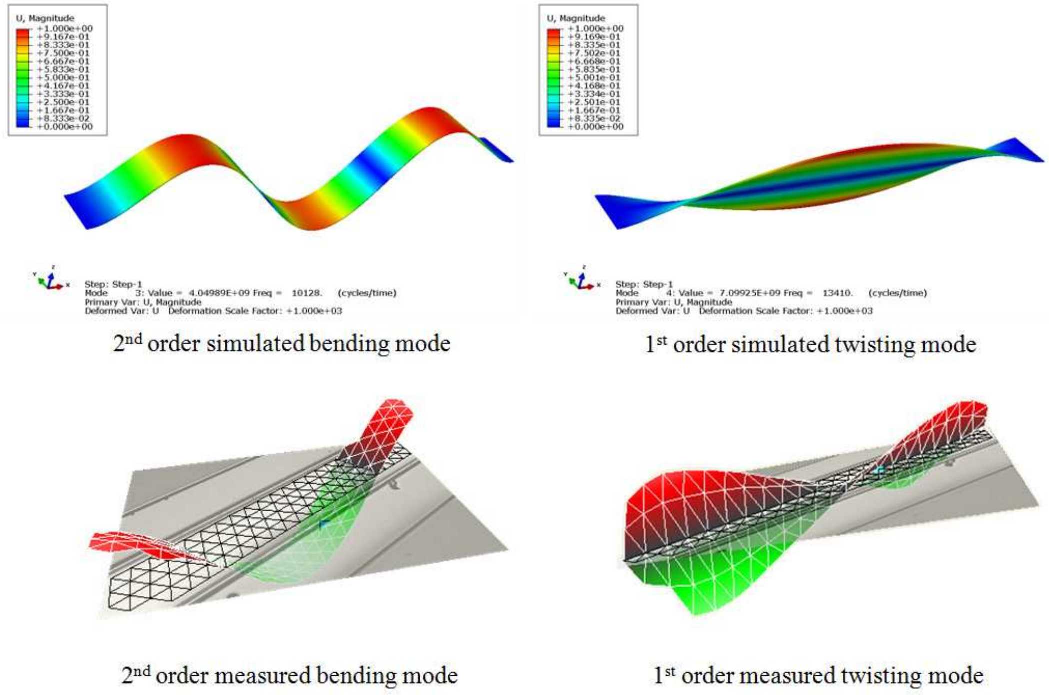

Figure S3. The Laser-Doppler Vibratory technology measured natural frequencies and the corresponding mode shapes of the "stiff" material AlN/Si based micro-belt (length $\times$ width $\times$ thickness: 10 mm $\times$ 1 mm $\times$ 21 μm), and the simulated natural frequencies and the corresponding mode shapes of it.

S2 Natrual vibration and wind driven fluttering of belts

S2.1 Bending and torsional vibrations of belts

S2.1.1 Classical beam theory

First, the vibration of belt under zero air velocity is studied. Assuming that the deformation of bending and torsion for the belt is small enough, and when it bends and twists naturally, the motion of it follows Euler-Bernoulli theory for bending and St. Venant theory for torsion[S1,S2]:

$$EI_x \frac{\partial^4 v}{\partial z^4} + m\frac{\partial^2 v}{\partial t^2} = 0, \tag{S2.1}$$

$$-GJ\frac{\partial^2 \varphi}{\partial z^2} + mK_m^2 \frac{\partial^2 \varphi}{\partial t^2} = 0, \tag{S2.2}$$

where x is along the flow direction, z is along the axis of beam or belt, y is the normal direction of the belt surface, and t is time. v is the deformation of belt (or beam) in the y direction, and φ is the twist angle of each element of belt. I_x is the second axial moment of area for belt about the x-direction. E is Young's modules, G is the shear modules, and m is the mass of the belt/beam per unit length, J is the torsional constant (stiffness), K_m is the radius of gyration, A is the cross section area. For the thin rectangular belts investigated in the current study, $J = \frac{Bh^3}{3}$ [S3].

S2.1.2 Beam with axial force

With an axial force inside the belt, the governing equation of bending vibration can be rewritten as[S4,S5]:

$$EI_x \frac{\partial^4 v}{\partial z^4} - T\frac{\partial^2 v}{\partial z^2} + m\frac{\partial^2 v}{\partial t^2} = 0, \tag{S2.3}$$

where T is the axial internal force, which is a constant for belts under extremely pre-stretching.

Similarly, the torsional vibration of belt with a constant internal axial force can be analyzed in the following way. Assuming the torsional angle φ at location z, then the angle θ (see Figure S4) between the deformed string in the belt and the original axis z is

$$\theta = r\frac{\partial \varphi}{\partial z}.$$

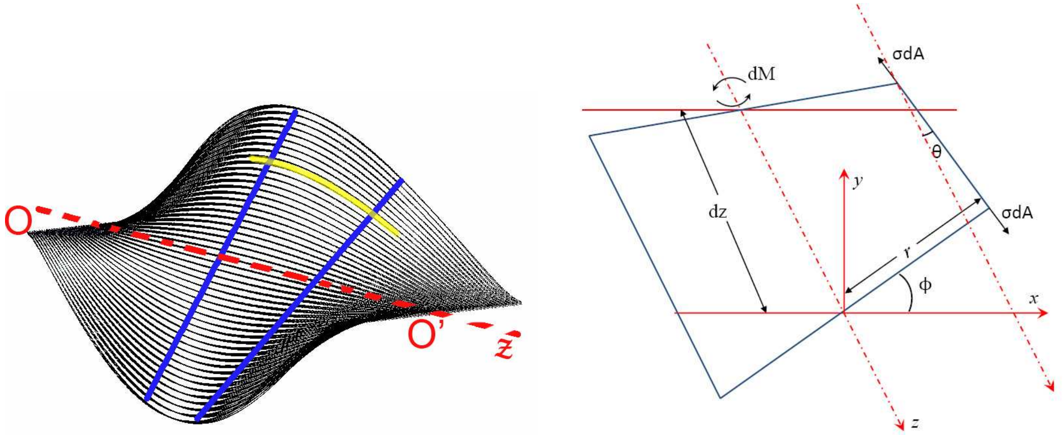

Figure S4. The schematic of the torsional deformation of the belt. Left hand side is the whole picture of belt which is divided into threads; right hand side is the section between the two blue lines and the internal stress in the thread.

Assuming the axial tension is uniform within the cross section of the belt, so it can be expressed in the form

$$\sigma = \frac{T}{A},$$

where A is the cross section area, and T is the total axial stretching force.

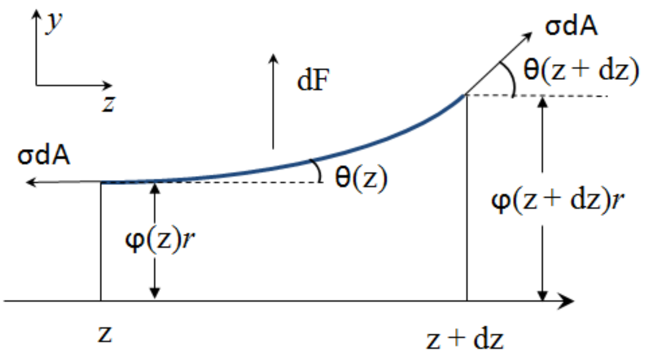

Figure S5. The schematic of the torsional deformation of a string inside the belt.

Similar to the string, the rotational force per unit length of belt (see Figure S5) due to the different deformation angle of axial string on the small cross section area dA is

$$dF = \sigma dA \frac{\partial \theta}{\partial z} = \frac{T}{A} dA \frac{\partial \theta}{\partial z} = \frac{T}{A} dA r \frac{\partial^2 \varphi}{\partial z^2}.$$

The torque of this force about the axis OO' (or z axis) is

$$dM = rdF = \frac{T}{A}\frac{\partial^2 \varphi}{\partial z^2} r^2 dA.$$

Integrate it over the cross section

$$M = \frac{T}{A}\frac{\partial^2 \varphi}{\partial z^2} \int r^2 dA = \frac{TI}{A}\frac{\partial^2 \varphi}{\partial z^2},$$

where I is the polar moment of area for rectangular beam defined as $I = \iint r^2 dA = \frac{B^3 h + Bh^3}{12}$. Then combing with torque due to the shear stress $GJ\frac{\partial^2 \varphi}{\partial z^2}$ ($J = \frac{Bh^3}{3}$ for rectangular thin belt in the current study[S3].), and considering the angular acceleration, the governing equation can be obtained as

$$-\left(GJ + \frac{TI}{A}\right)\frac{\partial^2 \varphi}{\partial z^2} + mK_m^2 \frac{\partial^2 \varphi}{\partial t^2} = 0, \tag{S2.4}$$

where m is the mass of belt per unit length, and K_m is the radius of gyration, and $mK_m^2 = I_\alpha$ is the moment of inertial.

S2.1.3 large deformation

However, when the bending deformation/deflection of the belt is large, the axial force will be affected by the associated axial force then. Assuming this axial force is still a constant along the axis, so the additional strain of the belt along the axis due to the bending deformation is

$$\varepsilon = \frac{\int_0^L \sqrt{dz^2 + dv^2} - L}{L} \approx \frac{1}{2L}\int_0^L \left(\frac{\partial v}{\partial z}\right)^2 dz.$$

Thus, the total axial force considering bending deformation of the belt becomes

$$T = T_0 + \frac{EA}{2L}\int_0^L \left(\frac{\partial v}{\partial z}\right)^2 dz, \tag{S2.5}$$

where T_0 is the pre-existing axial force due to pre-stretching. Replacing the axial force in Eqs. (S2.3) and (S2.4) by the total axial force in Eq. (S2.5) yields

$$EI_x \frac{\partial^4 v}{\partial z^4} - \left[T_0 + \frac{EA}{2L}\int_0^L \left(\frac{\partial v}{\partial z}\right)^2 dz\right]\frac{\partial^2 v}{\partial z^2} + m\frac{\partial^2 v}{\partial t^2} = 0, \tag{S2.6}$$

$$-\left\{GJ+\left[\frac{T_0}{A}+\frac{E}{2L}\int_0^L\left(\frac{\partial v}{\partial z}\right)^2 dz\right]I\right\}\frac{\partial^2\varphi}{\partial z^2}+mK_m^2\frac{\partial^2\varphi}{\partial t^2}=0. \tag{S2.7}$$

When $T_0L^2 \ll EI_x$, the bending stiffness is dominant, and then Eq. (S2.6) reduces to the theory of beam under large deformation given by Conway[S6,S7].

$$EI_x\frac{\partial^4 v}{\partial z^4}-\frac{EA}{2L}\int_0^L\left(\frac{\partial v}{\partial z}\right)^2 dz\frac{\partial^2 v}{\partial z^2}+m\frac{\partial^2 v}{\partial t^2}=0.$$

On the other hand, when $T_0L^2 \gg EI_x$, the pre-stretching force T_0 is dominant, and then Eq. (S2.6) reduces to the Kirchhoff equation for nonlinear vibration of strings[S8–S12].

$$-\left[T_0+\frac{EA}{2L}\int_0^L\left(\frac{\partial v}{\partial z}\right)^2 dz\right]\frac{\partial^2 v}{\partial z^2}+m\frac{\partial^2 v}{\partial t^2}=0.$$

S2.2 Degree of freedom (DOF) reduction for vibration of belts

S2.2.1 Small deformation

When the deformations of bending is very small $\frac{EA}{2L}\int_0^L\left(\frac{\partial v}{\partial z}\right)^2 dz \ll T_0$, Eqs. (S2.3) and (S2.4) can be used to solve the problem. They are general linear partial differential equations (PDEs) with constant parameters, applying the variable separation approach as following:

$$v=h(t)V(z), \tag{S2.8}$$

$$\varphi=\alpha(t)\Phi(z). \tag{S2.9}$$

Let $h(t)$ and $\alpha(t)$ respectively to be the bending deformation and torsional angle of belt/beam at a typical position along the axis (normally the maximum deformation along the axis), representing the features of vibration in time space. On the other hand, $V(z)$ and $\Phi(z)$ are the deformation distribution along the axis, representing vibration mode (as shown in Figs. S3–??). Then

$$EI_xh\frac{\partial^4 V}{\partial z^4}-T_0h\frac{\partial^2 V}{\partial z^2}+mV\frac{\partial^2 h}{\partial t^2}=0$$
$$\Rightarrow \frac{1}{V}\left(\frac{EI_x}{m}\frac{\partial^4 V}{\partial z^4}-\frac{T_0}{m}\frac{\partial^2 V}{\partial z^2}\right)=-\frac{1}{h}\frac{\partial^2 h}{\partial t^2}=\text{const},$$
$$-\left(GJ+\frac{T_0I}{A}\right)\alpha\frac{\partial^2\Phi}{\partial z^2}+mK_m^2\Phi\frac{\partial^2\alpha}{\partial t^2}=0$$
$$\Rightarrow \frac{1}{mK_m^2}\left(GJ+\frac{T_0I}{A}\right)\frac{\partial^2\Phi}{\Phi\partial z^2}=\frac{\partial^2\alpha}{\alpha\partial t^2}=\text{const}.$$

Thus, the ordinary differential equations (ODEs) can be obtained instead of the original partial differential equations (PDEs):

$$\ddot{h}+\omega_{h0}^2h=0, \tag{S2.10}$$

$$\ddot{\alpha}+\omega_{\alpha0}^2\alpha=0, \tag{S2.11}$$

where ω_{h0} and $\omega_{\alpha0}$ are the natural frequency of vibration for each mode of bending and torsion with

$$\begin{aligned}&EI_x\frac{\partial^4 V}{\partial z^4}-T_0\frac{\partial^2 V}{\partial z^2}-m\omega_{h0}^2V=0,\\&V=e^{\lambda_h\frac{z}{L}}\Rightarrow\frac{EI_x}{mL^4}\lambda_h^4-\frac{T}{mL^2}\lambda_h^2-\omega_{h0}^2=0,\\&V=0,V'=0\text{ at the belt ends,}\end{aligned} \tag{S2.12}$$

and for torsion

$$\left(GJ+\frac{T_0 I}{A}\right)\frac{\partial^2\Phi}{\partial z^2}+mK_m^2\omega_{\alpha 0}^2\Phi=0,$$
$$\Phi=e^{\lambda_\alpha \frac{z}{L}}\Rightarrow\frac{\left(GJ+\frac{T_0 I}{A}\right)}{mK_m^2L^2}\lambda_\alpha^2+\omega_{\alpha 0}^2=0, \tag{S2.13}$$
$$\Phi=0 \text{ at the belt ends.}$$

Thus, the vibration modes and respective frequency can be solved based on the boundary conditions of belts at its two ends. In the current study, this process is conducted in ABAQUS software.

S2.2.2 Large deformation

When the deformation of bending for the belt is large ($\frac{EA}{2L}\int_0^L\left(\frac{\partial v}{\partial z}\right)^2 dz$ is close or even higher than T_0), the nonlinear vibration equations Eqs. (S2.6) and (S2.7) need to be used to solve the problem. Although these two PDEs are nonlinear, the leading order of the vibration can be linearized and approximately described by

$$\ddot{h}+\omega_{h0}^2 h=0,$$

and

$$\ddot{\alpha}+\omega_{\alpha 0}^2\alpha=0,$$

where $h(t)$ and $\alpha(t)$ are also respectively the maximum deformation along the axis similar to the situation with small deformation, representing the features of vibration in time space. Speciafically, Eqs. (S2.6) and (S2.7) were solved numerically first, and then the key features of the leading order of the vibrations were extracted using Fast Fourier transform (FFT) in order to obtain ω_{h0} and $\omega_{\alpha 0}$.

S2.3 Analysis of 2DOF fluttering system

Thus, the ordinary differential equations (ODEs) for the deformations of bending and torsion at the typical position along the axis of belt (normally the maximum deformation along the belt's axis)

$$\ddot{h}+\omega_{h0}^2 h=0 \tag{S2.14}$$

and

$$\ddot{\alpha}+\omega_{\alpha 0}^2\alpha=0 \tag{S2.15}$$

are not only accurate for the vibrating belt with small deformation, but is also applicable for the leading order of belt's vibration with large deformation. Generally, there is friction and dissipation in the vibration system, bringing the damping effect into the governing equations. In addition, the external lift and torque due to the aerodynamic force are also added in, and then the governing equations are obtained for this two degree of freedom (2DOF) system[S13–S17]

$$m\left(\ddot{h}+2\zeta_{h0}\omega_{h0}\dot{h}+\omega_{h0}^2 h\right)=L\left(h,\alpha,\dot{h},\dot{\alpha}\right) \tag{S2.16}$$

and

$$I_\alpha\left(\ddot{\alpha}+2\zeta_{\alpha 0}\omega_{\alpha 0}\dot{\alpha}+\omega_{\alpha 0}^2\alpha\right)=M\left(h,\alpha,\dot{h},\dot{\alpha}\right), \tag{S2.17}$$

where ζ_{h0} and $\zeta_{\alpha 0}$ are the original damping ratio for bending and torsion without incoming flow, and $I_\alpha=mK_m^2$. L and M are the aerodynamic lift force and torque per unit length of belt respectively.

According to Scanlan and Theodorsen's theory[S13–S16], the lift force and torque is determined by the local motion of belt/beam as

$$L=\rho_a U^2 B\left[KH_1^*\frac{\dot{h}}{U}+KH_2^*\frac{B\dot{\alpha}}{U}+K^2H_3^*\alpha+K^2H_4^*\frac{h}{B}\right], \tag{S2.18}$$

$$M=\rho_a U^2 B^2\left[KA_1^*\frac{\dot{h}}{U}+KA_2^*\frac{B\dot{\alpha}}{U}+K^2A_3^*\alpha+K^2A_4^*\frac{h}{B}\right], \tag{S2.19}$$

where $K = B\beta/U$ is the non-dimensional frequency, β is the final fluttering frequency of belt, B is the belt width in the flow direction, U is the velocity of the coming air flow, and ρ_a is the density of air. Besides, H_i^* and A_i^* are the generalized flutter derivatives related to the cross section feature of the belt and the non-dimensional frequency K.

With substituting Eqs. (S2.18) and (S2.19) back into Eqs. (S2.16) and (S2.17) and rearranging the same order terms, it is obtained in matrix form that

$$\mathbf{M_e}\ddot{\mathbf{X}} + \mathbf{C_e}\dot{\mathbf{X}} + \mathbf{K_e}\mathbf{X} = \mathbf{0}, \quad \text{(S2.20)}$$

where $\mathbf{X} = \begin{bmatrix} h \\ \alpha \end{bmatrix}$. It can be further simplified into the following form that

$$\mathbf{R}\dot{\mathbf{y}} + \mathbf{S}\mathbf{y} = \mathbf{0}, \quad \text{(S2.21)}$$

where $\mathbf{R} = \begin{bmatrix} \mathbf{C_e} & \mathbf{M_e} \\ \mathbf{M_e} & \mathbf{0} \end{bmatrix}$, $\mathbf{S} = \begin{bmatrix} \mathbf{K_e} & \mathbf{0} \\ \mathbf{0} & -\mathbf{M_e} \end{bmatrix}$ and $\mathbf{y} = \begin{bmatrix} \mathbf{X} \\ \dot{\mathbf{X}} \end{bmatrix}$.

Assuming its solution is $\mathbf{y} = \Psi' e^{\lambda t}$, then we got $(\lambda \mathbf{R} + \mathbf{S})\Psi' = 0$ whose Eigen vector is and the Eigen-function is

$$\det(\lambda \mathbf{R} + \mathbf{S}) = 0 \quad \text{(S2.22)}$$

whose solutions are

$$\begin{aligned} \lambda &= -\zeta_i \omega_i \pm \sqrt{1-\zeta_i^2}\,\omega_i j \\ &= -\chi_i \pm \beta_i j \ (i = h, \alpha), \end{aligned} \quad \text{(S2.23)}$$

where j is complex number $\sqrt{-1}$, the subscript h is related to bending, and α is related to torsion. Solving such nonlinear eigenvalue problem needs an iterative process between Eqs. S2.18, S2.19 and S2.23 until β in them coincides. Normally, the mechanical dynamic matrix $\mathbf{R}^{-1}\mathbf{S}$ of a 2DOF system has four Eigen-values, which are two pairs of conjugates and show the features of the whole vibrating system. ω_i is the angular frequency of the new system, and χ_i represents the damping feature of the coupled vibrating system. When $\chi_i > 0$, the system is stable, the corresponding amplitude will damp to zero after long period; however, when $\chi_i < 0$, it is an unstable system, the corresponding amplitude will magnify by itself until it breaks down. Since the flutter derivatives are also dependent on the resultant vibration frequency, so solving the fluttering equations needs iterations between time-domain and frequency domain in order to obtain the correct flutter frequency and the corresponding damping ratio[S17].

Then the typical bending deformation and the torsion angle in the time-domain can be written as

$$h = A_{hh} e^{-\chi_h t} \cos(\beta_h t + \theta_{hh}) + A_{h\alpha} e^{-\chi_\alpha t} \cos(\beta_\alpha t + \theta_{h\alpha}) \quad \text{(S2.24)}$$

and

$$\alpha = A_{\alpha h} e^{-\chi_h t} \cos(\beta_h t + \theta_{\alpha h}) + A_{\alpha\alpha} e^{-\chi_\alpha t} \cos(\beta_\alpha t + \theta_{\alpha\alpha}) \quad \text{(S2.25)}$$

where χ_h and β_h corresponds to the damping factor and angular frequency due to bending, χ_α and β_α correspond to the damping factor and angular frequency due to torsion. When $\chi_i > 0$, the system with the corresponding vibration is stable at the specific frequency; however, when $\chi_i < 0$, it is an unstable system at the corresponding frequency. It can also be found that the both bending and torsional deformation can vibrate at two frequencies, of which one will finally dominate another (depending on the damping factor of each component).

The ratio between the bending and torsion can be determined by terms of Eigen-vector $\Psi' = \begin{bmatrix} h \\ \alpha \\ \dot{h} \\ \dot{\alpha} \end{bmatrix}$, so

$$\frac{A_{hi}}{A_{\alpha i}} = \frac{\|\Psi'_{1i}\|}{\|\Psi'_{2i}\|} (i = h, \alpha), \quad \text{(S2.26)}$$

where subscript 1 and 2 respectively means the first and second component of Eigen-vector, and $i = h$, α respectively means the Eigen-vector correlated to bending or torsion.

In addition, the final flutter frequency f_i and damping ratio ζ_i at different air incoming velocity can be derived as:

$$
\begin{aligned}
f_i &= \frac{\beta_i}{2\pi} \\
\omega_i &= \sqrt{\chi_i^2 + \beta_i^2} \\
\zeta_i &= \frac{\chi_i}{\omega_i} \; (i = h, \alpha).
\end{aligned}
\tag{S2.27}
$$

S2.4 Results for "stiff" material based on theoretical prediction

The above theory and the natural frequency obtained by ABAQUS (bending: 10128Hz, torsional: 13410Hz) are employed to calculate the vibration frequencies of belts based on "stiff" material and corresponding damping ratio as shown in Tab. S2 and Figure S6 in order to evaluate the onset of fluttering and flutter frequency. The vibration frequencies and damping ratios are grouped into two series: the torsional frequency, and the other is bending frequency. It shall be noted that bending and torsion motions contain two components which are associated to bending and torsional frequencies respectively (see Eqs. S2.24 and S2.25). It is observed that when the air flow velocity exceeds the critical value (75m/s herein), the torsion damping ratio ζ_α becomes negative, so the motion components associated to torsional frequency becomes divergent for both bending and torsional motions. In other words, the bending and torsional amplitude become larger and larger when the air velocity exceeds onset velocity, and so the flutter occurs.

Table S2. The mathematically predicted vibrating frequencies and damping ratios for flutter phenomenon.

U(m/s)	f_α(Hz)	ζ_α	f_h (Hz)	ζ_h
0	13410	0.001	10128	0.001
5	13405.38	0.001122	10135.24	0.003158
10	13391.44	0.001466	10143.94	0.005323
15	13367.99	0.002001	10154.22	0.007537
20	13334.83	0.002696	10166.26	0.009845
25	13291.68	0.003518	10180.29	0.012296
30	13238.19	0.00443	10196.55	0.014943
35	13173.96	0.005387	10215.39	0.01785
40	13098.46	0.006334	10237.21	0.021093
45	13011.08	0.007197	10262.53	0.024766
50	12911.06	0.007877	10291.98	0.028997
55	12797.56	0.008223	10326.3	0.033957
60	12669.74	0.008009	10366.3	0.039898
65	12527.15	0.006878	10412.52	0.047201
70	12370.99	0.004261	10464.26	0.056466
75	12207.24	-0.00064	10516.6	0.068569
80	12050.98	-0.00844	10555.04	0.084342
85	11921.04	-0.01854	10557.77	0.103436
90	11822.7	-0.02932	10515.53	0.124124
95	11748.18	-0.03967	10435.66	0.145083
100	11688.77	-0.0492	10327.58	0.165945
105	11638.81	-0.05794	10197.14	0.186782
110	11595.03	-0.06595	10045.82	0.208047
115	11555.56	-0.07335	9873.459	0.230175
120	11519.28	-0.08021	9680.519	0.253298

S3 Calculation of bending deformations of belt from the voltage output

S3.1 "Stiff" AlN/Si belt

The Euler-Bernoulli model s shown in Figure S7. The AlN and Si substrate both bend about a common neutral axis which is no longer the neutral axis of the belt. Perfect bonding is assumed, and the AlN is considered to be a layer of the belt. This neutral axis is calculated by a modulus-weighted algorithm.

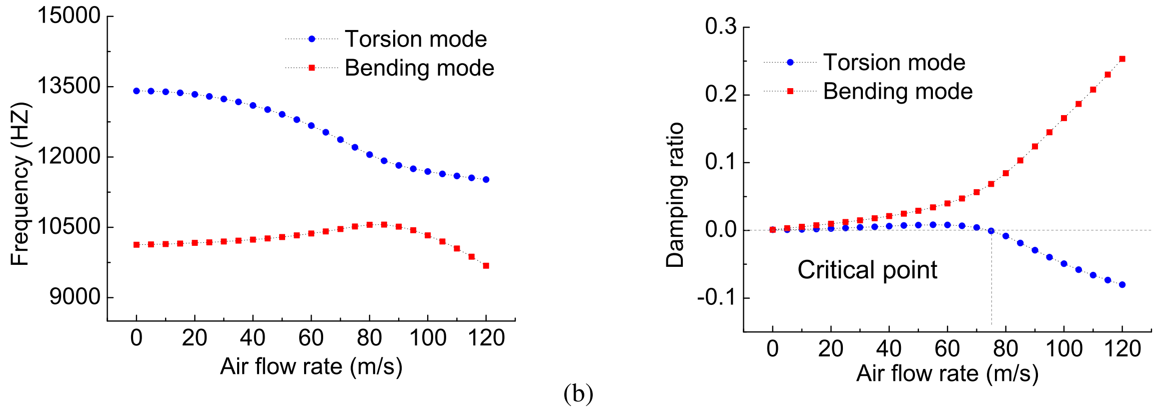

(a) (b)

Figure S6. The mathematic description of the flutter occurrence requirement for the 3rd order bending-1st order torsional mode pair to the "stiff" material AlN/Si based double clamped micro-belt (a) the flutter frequency vs. air flow velocity; (b) the damping ratio vs. air flow velocity.

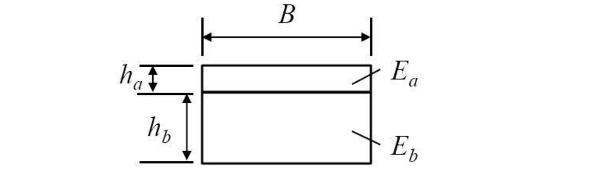

Figure S7. The schematic of Euler Bernoulli model of "stiff" AlN/Si belt.

The equation for the distance to the neutral axis can be written as

$$y_s = \frac{\frac{h_a}{2} h_a \frac{E_a}{E_b} + (h_a + \frac{h_b}{2}) h_b}{h_a \frac{E_a}{E_b} + h_b}, \tag{S3.1}$$

where E_a, E_b are the Young's Modulus of the AlN and Si belt; h_a, h_b are the thickness of the AlN and Si belt.

To simplify the calculations, the average strain in the AlN is determined and used to find the voltage. The average strain in AlN ε_a is

$$\varepsilon_a = -\frac{M}{(E_a I_a + E_b I_b)}(y_s - \frac{h_a}{2}), \tag{S3.2}$$

where

$$I_a = \int_{y_s - h_a}^{y_s} B y^2 dy = \frac{1}{3} B [y_s^3 - (y_s - h_a)^3], \tag{S3.3}$$

$$I_b = \int_{-(h_a + h_b - y_s)}^{y_s - h_a} B y^2 dy = \frac{1}{3} B [(h_a + h_b - y_s)^3 + (y_s - h_a)^3], \tag{S3.4}$$

and M is the bending moment of the belt. So

$$\varepsilon_a = -\frac{6 M E_b h_b (h_a + h_b)}{B[E_a^2 h_a^4 + E_b^2 h_b^4 + 2 E_a h_a E_b h_b (2h_a^2 + 3 h_a h_b + 2 h_b^2)]}. \tag{S3.5}$$

The voltage on the AlN surfaces is related to the stress by

$$V = g_{31} h_a \sigma_a = g_{31} h_a E_a \varepsilon_a, \tag{S3.6}$$

where g_{31} is the AlN voltage constant and σ_a is stress in AlN. Finally, the voltage output voltage can be obtained as

$$V = -\frac{6 g_{31} M \Psi (1+T)}{B h_a [1 + \Psi^2 T^2 + 2\Psi(2 + 3T + 2T^2)]}, \tag{S3.7}$$

where $\Psi = \frac{E_b h_b}{E_a h_a}$, $T = \frac{h_b}{h_a}$.

The maximum bending deformation of the AlN belt is

$$C_v \approx l \sqrt{(1 + \varepsilon_a)^2 - 1}. \tag{S3.8}$$

S3.2 "Flexible" P(VDF-TrFE) belt

The total charge generated from the deformation induced by the vibration of the P(VDF-TrFE) (see Figure S9) can be obtained by the equation:

$$Q = CV, \tag{S3.9}$$

where $C = \frac{\varepsilon_0 \varepsilon_r A_p}{h_p}$ is the capacitance of the P(VDF-TrFE) belt, in Farads; $A_p = B_p \times l_p$ is the surface area of the P(VDF-TrFE) belt on the top side; ε_r is the relative static permittivity of the P(VDF-TrFE) (it is 9 for the P(VDF-TrFE) material); ε_0 is the electric constant ($\varepsilon_0 \approx 8.854 \times 10^{-12} F/m$); h_p is the thickness of the P(VDF-TrFE) belt; l_p is the length of the P(VDF-TrFE) belt; E_p is the Young's Modulus of the P(VDF-TrFE) belt.

The force applied on the belt is

$$F = \frac{Q}{d_{31}},$$

where d_{31} is the piezoelectric coefficient ($6 \times 10^{-12} C/N$) of the P(VDF-TrFE) thin film.

Since the strain generated is

$$\varepsilon_p = \frac{F}{E_P A_p} = \frac{CV}{d_{31} E_P A_p},$$

hence the maximum bending deformation of the P(VDF-TrFE) belt is

$$h_p \approx l_p \sqrt{(1 + \varepsilon_p)^2 - 1}. \tag{S3.10}$$

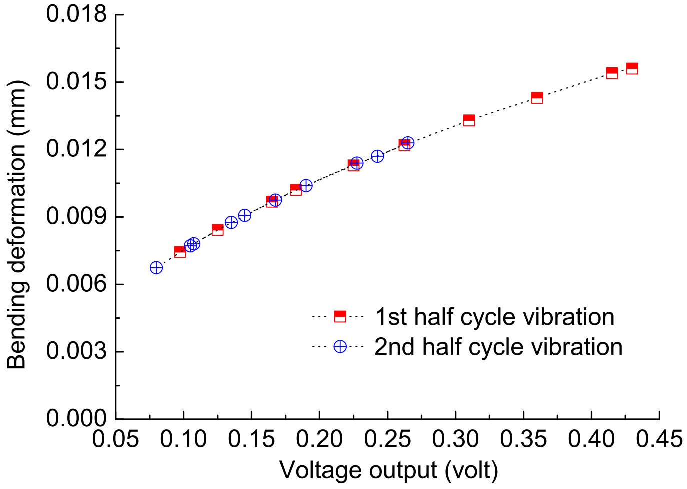

Figure S8. The correlation between voltage output and bending deformation for the AlN/Si belt, and the range of bending deformation in the experiment.

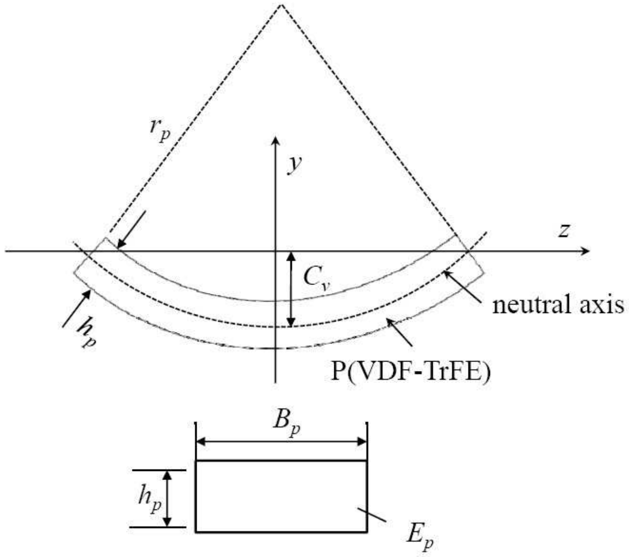

Figure S9. The schematic of deformation of "flexible" P(VDF-TrFE) belt.

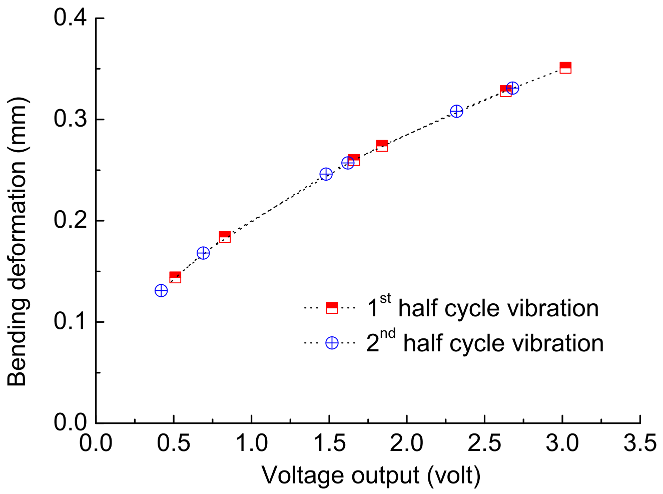

Figure S10. The correlational between voltage output and bending deformation for the P(VDF-TrFE) belt, and the range of bending deformation in the experiment.

S4 Effect of Deformation amplitude on natural frequency of torsion

S4.1 "Stiff" AlN/Si belt

The vibration of "stiff" belt is dominated by the bending stiffness and torisional stiffness due to shear stress, and there is no axial pre-stretching force inside the belt. So Eqs. (S2.6) and (S2.7) under $T_0 = 0$ is solved with the initial condition of second order perturbation are given as

$$v|_{t=0} = C_v \cos\left(\frac{n\pi z}{L}\right), \dot{v}|_{t=0} = 0,$$
$$\varphi|_{t=0} = C_\varphi \cos\left(\frac{n\pi z}{L}\right), \dot{\varphi}|_{t=0} = 0, \tag{S4.1}$$

where n stands for the number of mode, together with boundary condition, and C_v and C_φ are the maximum bending deformation and torsional angle respectively. The boundary condition is

$$v = 0, v' = 0 \text{ at the belt ends,}$$
$$\varphi = 0 \text{ at the belt ends.}$$

The numerical results shown in Tab. S3 demonstrate the pair of peak frequencies which are related to the fluttering phenomenon observed in the experiment. It is found that the nonlinear effect of deformation under 0.01mm (observed in the experiment) is ignorable: the torsional frequency is almost a constant within the different deformation range; the bending frequency slightly increases a little. Thus, it is natural that the final fluttering frequency is almost a constant at different incoming air velocity, with considering the fact found that the final fluttering frequency is slightly lower than the torsional frequency. Thus it can be concluded that linear vibration equations Eqs. (S2.3) and (S2.4) are still applicable for the vibration of "stiff" AlN/Si belts vibrating at small deformation in the current experiment.

Table S3. The mathematic model retrieved natural frequencies of the bending and torsional modes and the corresponding flutter frequency f_F for "stiff" AlN/Si based micro-belt.

C_v (mm)	f_{h0}(Hz)	$f_{\alpha 0}$(Hz)	f_F(Hz)
1×10^{-3}	10128	13410	12207
2×10^{-3}	10128	13410	12238
5×10^{-3}	10395	13410	12269
1×10^{-2}	10928	13410	12300

S4.2 "Flexible" P(VDF-TrFE) belt

In contrast of "stiff" AlN/Si belt, the vibration of "flexible" belt is dominated by the stretching force. So Eqs. (S2.6) and (S2.7) under $T_0 = \text{constant} = 0.07N$ is solved with the initial condition of second order perturbation are given as

$$\begin{aligned} v|_{t=0} &= C_v \cos\left(\frac{n\pi z}{L}\right), \dot{v}|_{t=0} = 0, \\ \varphi|_{t=0} &= C_\varphi \cos\left(\frac{n\pi z}{L}\right), \dot{\varphi}|_{t=0} = 0, \end{aligned} \tag{S4.2}$$

together with the boundary condition

$$v = 0, v' = 0 \text{ at the belt ends,}$$
$$\varphi = 0 \text{ at the belt ends,}$$

where n stands for the number of mode, together with boundary condition, and C_v and C_φ are the maximum bending deformation and torsional angle respectively.

The numerical results are shown in Tab. S4, and they only demonstrate the one pair of peak frequencies which are related to the fluttering phenomenon observed in the experiment. It is found that the bending deformation of belt about 0.1–0.4 mm (recorded in the experiment) has highly nonlinear effect on both bending and torsional frequencies. Thus, with considering the fact that the final fluttering frequency is determined by the torisional frequency, it is unsurprising that the final fluttering frequency dramatically increases with the increasing incoming air velocity, which is consistent with the experimental observations. It is concluded that for bending and torsional vibration for belts with such large deformation, nonlinear vibration equations Eqs. (S2.6) and (S2.7) shall be used to involve nonlinearity in order to obtain the accurate peak bending and torsional frequencies, then they can be used to calculate the final fluttering frequency based the 2DOF Theodorsen theory (see §S2).

Table S4. The mathematic model retrieved natural frequencies of the bending and torsional modes and the corresponding flutter frequency f_F for less pre-stressed "flexible" P(VDF-TrFE) micro-belt.

C_v (mm)	f_{h0} (Hz)	$f_{\alpha 0}$ (Hz)	f_F (Hz)
1×10^{-3}	2850	3350	3300
1×10^{-1}	2900	3400	3370
2×10^{-1}	3100	3500	3490
3×10^{-1}	3400	3650	3645
4×10^{-1}	3750	3800	3797

If the pre-existing stretching force is increased up to $T_0 = \text{constant} = 0.21N$, then the relative nonlinearity of large deformation becomes much lower than that in the case with smaller stretching force $T_0 = \text{constant} = 0.07N$. Similar numerical simulation was conducted, and the results are listed in Tab. S5.

Table S5. The mathematic model retrieved natural frequencies of the bending and torsional modes and the corresponding flutter frequency f_F for more pre-stressed "flexible" P(VDF-TrFE) micro-belt.

C_v (m)	f_{h0} (Hz)	$f_{\alpha 0}$ (Hz)	f_F (Hz)
1×10^{-3}	4775	5050	5000
1×10^{-1}	4800	5050	5020
2×10^{-1}	4925	5125	5115
3×10^{-1}	5125	5175	5170
4×10^{-1}	5375	5375	5372

References

[S1] Timoshenko, S. *History of strength of materials: with a brief account of the history of theory of elasticity and theory of structures* (Courier Corporation, 1953).

[S2] Dokumaci, E. An exact solution for coupled bending and torsion vibrations of uniform beams having single cross-sectional symmetry. *Journal of Sound and Vibration* **119**, 443–449 (1987).

[S3] Ugural, A. C. & Fenster, S. K. *Advanced strength and applied elasticity* (Pearson education, 2003).

[S4] Truesdell, C. Outline of the history of flexible or elastic bodies to 1788. *The Journal of the Acoustical Society of America* **32**, 1647–1656 (1960).

[S5] Karabalin, R. B., Villanueva, L., Matheny, M., Sader, J. E. & Roukes, M. L. Stress-induced variations in the stiffness of micro-and nanocantilever beams. *Physical review letters* **108**, 236101 (2012).

[S6] Conway, H. The large deflection of simply supported beams. *The London, Edinburgh, and Dublin Philosophical Magazine and Journal of Science* **38**, 905–911 (1947).

[S7] Nishawala, V. *A study of large deflection of beams and plates*. Ph.D. thesis, Rutgers University-Graduate School-New Brunswick (2011).

[S8] Kirchhoff, G. *Vorlesungen über Mathematische Physik: Mechanik* (B.G. Teubner, 1876).

[S9] Carrier, G. On the nonlinear vibration of an elastic string. *Q. appl. Math.* **3**, 157–165 (1945).

[S10] Carrier, G. A note on the vibrating string. *Quart. Appl. Math* **7**, 101 (1949).

[S11] Narasimha, R. Non-linear vibration of an elastic string. *Journal of Sound and Vibration* **8**, 134–146 (1968).

[S12] Chen, L.-Q. & Ding, H. Two nonlinear models of a transversely vibrating string. *Archive of Applied Mechanics* **78**, 321–328 (2008).

[S13] Theodorsen, T. & Mutchler, W. General theory of aerodynamic instability and the mechanism of flutter. *NACA Report No. 496* (1935).

[S14] Scanlan, R. & Sabzevari, A. Experimental aerodynamic coefficients in the analytical study of suspension bridge flutter. *Journal of Mechanical Engineering Science* **11**, 234–242 (1969).

[S15] Scanlan, R. H. & Tomo, J. Air foil and bridge deck flutter derivatives. *Journal of Soil Mechanics & Foundations Div* (1971).

[S16] Scanlan, R. The action of flexible bridges under wind, i: flutter theory. *Journal of Sound and Vibration* **60**, 187–199 (1978).

[S17] Sarkar, P. P., Jones, N. P. & Scanlan, R. H. System identification for estimation of flutter derivatives. *Journal of Wind Engineering and Industrial Aerodynamics* **42**, 1243–1254 (1992).
